# Validation of Opportunities to Strengthen the National Disaster Medical System: The Military–Civilian NDMS Interoperability Study Quantitative Step

**DOI:** 10.1089/hs.2023.0051

**Published:** 2023-07-18

**Authors:** Thomas D. Kirsch, Clark J. Lee, David B. King, Adeteju A. Adeniji, Reena Sethi, Eric C. Deussing

**Affiliations:** Thomas D. Kirsch, MD, MPH, is a retired Professor and former Director, the National Center for Disaster Medicine and Public Health, Uniformed Services University of the Health Sciences. Bethesda, MD.; Eric C. Deussing, MD, MPH, is an Assistant Professor and National Disaster Medical System Pilot Program Director, the National Center for Disaster Medicine and Public Health, Uniformed Services University of the Health Sciences, Bethesda, MD.; Clark J. Lee, JD, MPH, is a Senior Research Associate, the National Center for Disaster Medicine and Public Health, Uniformed Services University of the Health Sciences and The Henry M. Jackson Foundation for the Advancement of Military Medicine, Inc., Bethesda, MD.; Adeteju A. Adeniji, MPH, is a Research Coordinator, the National Center for Disaster Medicine and Public Health, Uniformed Services University of the Health Sciences and The Henry M. Jackson Foundation for the Advancement of Military Medicine, Inc., Bethesda, MD.; Reena Sethi, DrPH, MHS, is a Senior Public Health Lead Researcher, National Center for Disaster Medicine and Public Health, Uniformed Services University of the Health Sciences and The Henry M. Jackson Foundation for the Advancement of Military Medicine, Inc., Bethesda, MD.; David B. King, PhD, is a Senior Biostatistician, The Henry M. Jackson Foundation for the Advancement of Military Medicine, Inc., Bethesda, MD.

**Keywords:** Hospital preparedness/response, Public health preparedness/response, Medical surge, Mass casualty care, Medical management/response, National Disaster Medical System

## Abstract

The National Disaster Medical System (NDMS) Pilot Program was authorized by Congress to improve the interoperability, capabilities, and capacity of the NDMS. To develop a roadmap for planning and research, the mixed methods Military–Civilian NDMS Interoperability Study (MCNIS) was conducted in 2020-2021. The initial qualitative phase of the study identified critical themes for improvement: (1) coordination, collaboration, and communication; (2) funding and incentives to increase private sector preparedness; (3) staffing capacity and competencies; (4) clinical and support surge capacity; (5) training, education, and exercises between federal and private sector partners; and (6) metrics, benchmarks, and modeling to track NDMS performance. These qualitative findings were subsequently refined, validated, and prioritized through a quantitative survey. Expert respondents ranked 64 statements based on weaknesses and opportunities identified during the qualitative phase. Data were collected using Likert scales, and multivariate proportions and confidence intervals were estimated to compare and prioritize each statement's level of support. Pairwise tests were conducted for each item-to-item pair to determine statistically significant differences. The survey results corroborated the earlier qualitative findings, with all weaknesses and opportunities ranked as important by a majority of respondents. Survey results also pointed to specific priorities for interventions within the 6 previously identified themes. As with the qualitative study, the survey found that the most common weaknesses and opportunities were related to coordination, collaboration, and communication, especially regarding information technology and planning at the federal and regional levels. These priority interventions are now being developed, implemented, and validated at 5 pilot partner sites.

## Introduction

The National Disaster Medical System (NDMS) was created in 1984 to improve the capacity of the US healthcare system to care for a surge of patients in the event of a large-scale military conflict or disaster.^1-3^ The US Department of Health and Human Services (HHS), under the Administration for Strategic Preparedness and Response (ASPR, previously the Office of the Assistant Secretary of Preparedness and Response), leads the NDMS in cooperation with the departments of Defense (DOD), Veterans Affairs, and Homeland Security. The NDMS has 3 main components: a network of definitive care hospitals, patient transportation capabilities, and medical response teams.^4^ The NDMS definitive care hospital component has rarely been activated at a large scale and never for a military conflict.^3,5^

Since its inception, there have been calls to improve the NDMS definitive care component. In 1995, a survey of over 100 emergency preparedness professionals found that most believed there was insufficient capacity or capability to provide healthcare after a catastrophic disaster involving 30,000 casualties.^6^ The 2003 SARS epidemic in Canada further demonstrated the fragility of healthcare systems in general,^7^ but it was the collapse of the New Orleans healthcare system and the poor healthcare response after Hurricane Katrina that led to urgent calls in the United States to reform the NDMS definitive care component.^5,8^ As Toner et al stated in 2007, “[c]urrent federal plans to provide medical care to the sick and injured in a large-scale mass casualty disaster are outdated and inadequate, putting the American public at great risk.”^9^ These concerns were again reinforced by the impact of Hurricane Sandy on the New York City healthcare system in 2012^10^ and the severe limitation of hospital capacity identified during surges in the COVID-19 pandemic.^11-13^

In December 2019, Congress recognized the need to strengthen NDMS's definitive care component and directed the Secretary of Defense to conduct an NDMS pilot program to increase the nation's medical surge capability, capacity, and interoperability.^14^ Given the DOD's leading role in the pilot and the NDMS's original purpose of preparing for casualties from a conventional military conflict in Europe,^1-3^ the scenario chosen to drive assessments and interventions for the pilot was an overseas conflict leading to 1,000 injured soldiers daily for 100 days. Before starting the pilot, an exploratory research study—the Military–Civilian NDMS Interoperability Study (MCNIS)—was conducted to identify specific NDMS weaknesses and opportunities to direct future pilot activities. MCNIS included qualitative and quantitative phases. The qualitative study used semistructured facilitated discussions with key NDMS federal and civilian stakeholders to identify critical NDMS challenges broadly. Six major themes emerged from the analysis: (1) coordination, collaboration, and communication; (2) funding; (3) staffing; (4) surge capacity; (5) training, education, and exercises; and (6) metrics, benchmarks, and modeling.^15^

This article reports on the results of a quantitative survey to refine, validate, and prioritize the MCNIS qualitative themes and to identify specific interventions needed to improve the NDMS definitive care component.

## Methods

### Survey Development and Administration

The weakness and opportunities identified during the initial MCNIS qualitative study were analyzed, organized, and translated into quantifiable statements designed to produce meaningful and actionable data to address the foundational MCNIS research question, “How can the capacities and capabilities of NDMS definitive care be improved in the context of a large, overseas military conflict?” After the statements were formulated, 8 nationally recognized NDMS subject matter experts and a survey method expert external to the research team reviewed each statement and provided suggestions relating to its clarity (“Does the statement make sense?”), readability (“Is the statement easy to read and understand?”), and content (“Is the statement relevant to the subject matter we're exploring?”). The final survey, consisting of 64 statements, was administered online using Max Survey (https://survey.max.gov) in September 2021.

Respondents ranked each statement using a 5-point Likert response format and positive and negative frames (ie, weaknesses and recommended opportunities) to prevent bias toward agreement. For statements related to weaknesses, survey respondents were asked to indicate the level of priority they would assign for the need to correct the identified concern using a 5-point Likert scale (1 is the lowest priority to 5 is the highest priority). Survey participants were asked to indicate their degree of support or opposition for statements related to opportunities using a 5-point Likert scale (1 is strongly oppose to 5 is strongly support).

Respondents were recruited using purposive and snowball sampling methods from either NDMS-participating federal agencies, NDMS-participating hospitals, or state or local emergency managers from NDMS Federal Coordinating Center (FCC) regions. Forty-nine respondents from the MCNIS qualitative study were invited initially. Another 43 respondents from a pilot stakeholder meeting were also recruited to participate in the survey. These individuals were asked to invite additional individuals with expertise in NDMS and healthcare emergency management.

The Human Research Protections Program Office for the Uniformed Services University of the Health Sciences determined that this study protocol did not require Institutional Review Board review under relevant federal regulations and DOD policy guidance on protecting human research subjects.

### Data Analysis

Survey respondents were grouped into 3 respondent types: FCC staff, federal interagency staff, and civilian/private sector staff. Only statements relevant to all 3 respondent types were included in the analysis, resulting in 14 NDMS weakness and 28 opportunity statements. The objectives of the data analysis were to: (1) prioritize the recommended opportunities and level of support for policy/action, as well as to address concerns/weaknesses as identified by the NDMS stakeholders; (2) determine whether there were statistically significant differences between the statements for the weaknesses and opportunities according to the 5-point Likert scale survey responses; and (3) determine if there was an interaction effect of responses among the 3 respondent types.

Because the survey responses were on a 5-point Likert scale, a 5-level multivariate proportion was estimated with the levels 1 to 5 corresponding from “strongly oppose” to “strongly support.” The 5 predicted proportions for each item and the upper and lower 95% predictive intervals for each were estimated. Agresti-Coull interval confidence limits were used in estimating the levels of strong support and support. Because the strong support level showed the most differentiation between categories, the strong support level was used to make each comparison. When comparing the proportion of strong support between statements within a given question category, Agresti-Caffo interval confidence limits were used to compare the difference in proportions.

To determine if the Likert scale proportions were correlated with the respondent type, proportional odds logistic regression models were fitted to the survey data. These models used the 5-point Likert scale responses as the outcome variable and respondent type as the explanatory predictor variable.

## Results

Eighty-seven of 147 (59%) invited respondents completed the survey. Of those 87 respondents, 19 (22%) worked at an FCC, 31 (36%) at the federal interagency level, and 37 (42%) were civilian/private sector (eg, healthcare, public health, emergency management, emergency medical services). There were no significant differences in the rankings of either weaknesses or opportunities by respondent type.

### Weaknesses

Table 1 lists 14 NDMS definitive care weaknesses ranked by respondents from lowest to highest. All 14 weaknesses were ranked greater than 50% “highest” and “high priority” combined (range 62% to 88%), with 4 (29%) ranked 80% or higher. None were ranked more than 5% “lowest priority,” and only 4 (29%) were ranked more than 10% “lowest” and “low priority” combined (range 1% to 14%). No significant differences were identified across the weaknesses and with pairwise comparisons within specific themes. The weaknesses relating to current information technology (IT) systems were ranked highest (88%), followed by lack of overall federal planning (85%), insufficient capacity to transport patients (84%), and lack of surge capacity in private sector hospitals (80%). Each identified weakness had at least 1 recommended solution/opportunity, and most had multiple (range 1 to 6). The respondent type was not a significant predictor of the proportion of support for any statement, as the *P* values for the variable were greater than .05 for every policy, meaning that respondent type could be dropped from the proportional odds logistic regression model.

**Table 1.** Weaknesses Ranked From Lowest to Highest Importance

Theme	Weakness	Lowest	Low	Routine	High	Highest	High and Highest
CCC (IT)	IT systems are inadequate to coordinate patient care across the federal and private sectors	0%	1%	11%	37%	51%	88%
CCC	An overall federal management plan to coordinate care across the federal and private sector does not exist	0%	2%	13%	38%	47%	85%
Surge capacity (patient movement)	Insufficient capacity and capability to move patients to appropriate care sites	0%	2%	14%	38%	46%	84%
Surge capacity (clinical)	NDMS private-sector hospitals have insufficient surge capacity	1%	3%	16%	37%	43%	80%
Funding	Insufficient reimbursement of private sector healthcare to care for NDMS patients over an extended period	0%	8%	22%	33%	37%	70%
CCC	FCCs are not adequately prepared to optimize patient distribution to appropriate care sites	0%	0%	31%	40%	29%	69%
TEE	Homeland security exercises and evaluations do not adequately address a combat medical surge scenario	2%	3%	28%	39%	27%	66%
Staffing	Private sector healthcare lacks sufficiently trained staff to respond to a national-level medical surge	2%	11%	28%	35%	23%	58%
CCC	Lack of planning for discharge, return-to-duty, rehabilitation, and return home for military patients in private sector healthcare facilities	2%	6%	34%	36%	22%	58%
TEE	Private sector hospital leaders may not be aware they are NDMS partners	3%	8%	27%	42%	20%	62%
Surge capacity (clinical)	Inadequate specialty care sites (eg, mental health, rehabilitation, burn centers) for large patient volume	0%	6%	36%	39%	19%	58%
Staffing	Private healthcare lacks flexibility to move staff	5%	9%	34%	35%	17%	52%
CCC	Lack of local government (eg, emergency management, public health) involvement in NDMS	0%	3%	36%	44%	17%	61%
CCC	Limited success in establishing partnerships between FCCs and local emergency management	2%	9%	28%	46%	16%	62%

Note: Percentages in table reflect the proportion selecting the Likert scale response out of a total of N = 87 respondents.

Abbreviations: CCC, coordination, collaboration, and communication; FCC, Federal Coordinating Center; IT, information technology; NDMS, National Disaster Medical System; TEE, training, education, and exercises.

### Recommended Opportunities

Table 2 lists the 28 recommended opportunities to improve the NDMS definitive care component, ranked by all respondent types from lowest to highest priority. All 28 opportunities were ranked as either “strongest” or “strong” priorities by at least 70% of respondents, with 11 (39%) ranked 90% or above (range 72% to 98%). None received more than 10% “lowest” or “low” priority ranking (range 0% to 9%). No significant ranking differences were identified across all opportunities, but with pairwise comparisons within specific themes, “staffing” had a single significant difference (ie, emergency credentialing was ranked higher than using Medical Reserve Corps providers, *P*<.05). IT systems to bridge the federal and private sectors were identified as 3 of the most important opportunities to improve the NDMS (98% for shareable electronic health records, 94% for patient tracking systems, and 92% for using telehealth to increase surge capacity). Increasing outreach to private sector healthcare leadership to build trust in NDMS partnerships was recommended by 95% of respondents. Improving how the Military Health System and FCCs distribute patients across federal and private sector healthcare facilities was recommended by 94%.

**Table 2.** Recommended Opportunities for Activities, Ranked From Strongest Opposition to Strongest Support

Theme	Opportunity	Strongly Oppose	Oppose	Neutral	Support	Strongly Support	Support and Strongly Support
CCC (IT)	Patient health records available across the federal and private sector	0%	0%	2%	31%	67%	98%
TEE	Increase outreach to private sector healthcare leadership to build trust in NDMS partnership	1%	2%	3%	32%	62%	95%
CCC (IT)	IT system to track patients across the federal and private sector	0%	2%	3%	26%	68%	94%
CCC	Improve how the Military Health System and FCCs distribute patients across federal and private sector healthcare	0%	1%	5%	31%	63%	94%
CCC	Conduct federal–private sector combat medical surge planning	0%	0%	8%	41%	52%	93%
Staffing	Give the federal government the ability to grant private healthcare NDMS providers emergency credentials and license portability	1%	1%	6%	34%	58%	92%
CCC (IT)	Use telehealth to increase patient care capacity	0%	2%	6%	38%	54%	92%
TEE	Conduct federal–private sector training, education, and exercises for combat medical surge	0%	1%	8%	40%	52%	92%
Funding	More robust options to reimburse for NDMS patient care	0%	0%	10%	32%	58%	90%
MBM	Develop NDMS operational modeling for healthcare preparedness and response	0%	0%	10%	41%	49%	90%
TEE	Develop NDMS just-in-time training for private sector hospitals	0%	1%	9%	48%	42%	90%
Surge capacity (clinical)	Include care sites exclusive to medical specialty (eg, mental health, rehabilitation, burn centers) as NDMS partners	0%	3%	8%	43%	46%	89%
Funding	Federal funding for federal and private healthcare surge capacity	0%	0%	12%	25%	63%	88%
Surge capacity (patient movement)	Increase transportation capacity from FCCs to NDMS hospitals	0%	0%	13%	31%	56%	87%
CCC	Develop regional healthcare bodies to coordinate patient distribution and care	1%	1%	11%	26%	60%	86%
Surge capacity	Conduct surge capacity assessments at federal and private healthcare facilities	0%	3%	13%	40%	44%	84%
CCC	Address the unique needs of military patients in private sector healthcare facilities	0%	2%	14%	40%	44%	84%
MBM	Develop a set of NDMS patient care preparedness metrics for the federal and private sectors	1%	1%	14%	41%	43%	84%
Staffing	Develop policies to provide for the movement of private sector healthcare staff to other facilities/regions	2%	7%	11%	36%	44%	80%
Funding	Fund regional healthcare coordinating entities for command and control	2%	5%	12%	37%	43%	80%
TEE	Develop EMS training, education, and exercises specific to NDMS patients	0%	2%	19%	37%	42%	79%
Surge capacity (clinical)	Include outpatient ambulatory care sites as NDMS patient care partners	0%	4%	17%	47%	32%	79%
TEE	Rotate military clinical providers through private sector NDMS hospitals to enhance training	0%	3%	19%	36%	42%	78%
CCC	Implement a single, standardized memorandum of agreement for nonhospital providers (eg, long-term care, skilled nursing)	0%	3%	19%	36%	42%	78%
TEE	Develop NDMS just-in-time training for FCC staff	0%	1%	14%	46%	39%	75%
TEE	Develop NDMS just-in-time training for the military	0%	1%	14%	43%	41%	74%
Staffing	Use the Medical Reserve Corps to provide clinical care	2%	2%	23%	33%	39%	72%
Surge capacity (clinical)	Include nonhospital inpatient providers (eg, long-term care, skilled nursing) as NDMS patient care partners	0%	6%	13%	46%	36%	72%

Note: Percentages in table reflect the proportion selecting the Likert scale response out of a total of N = 87 respondents.

Abbreviations: CCC, coordination, collaboration, and communication; EMS, emergency medical services; IT, information technology; MBM, metrics, benchmarks, and modeling; NDMS, National Disaster Medical System; TEE, training, education, and exercises.

## Discussion

This survey validated the results of the MCNIS qualitative study, with all weaknesses and opportunities ranked as important by a majority of respondents. It also pointed to specific priorities for interventions within each of the 6 previously identified themes. No significant differences were identified across all weaknesses or opportunities, and there were no interaction effects between weaknesses and opportunities with respondent type.

As with the qualitative study, the most common weaknesses identified were related to the theme of coordination, collaboration, and communication between federal, state, and local governments and private sector healthcare systems. These priority weaknesses focused on IT and planning at the federal and multijurisdiction regional levels. The 3 IT areas identified by the survey were: (1) tracking patients from the point of injury through the continuum of care to discharge, (2) having the ability to share medical records to coordinate care across multiple providers, and (3) using telehealth to expand surge capacity. These concerns are not new, as IT systems have been repeatedly recognized as critical components of healthcare disaster response coordination,^16-18^ and the need to improve healthcare IT systems for response has been recommended since at least Hurricane Katrina in 2005.^8^ IT interoperability is even more complicated when caring for military patients because of the disconnect between federal healthcare IT systems and those commonly used by private sector healthcare and emergency management entities.^19^

To address the problem of sharing health information, Congress passed the 2009 Health Information Technology for Economic and Clinical Health Act^20^ requiring HHS to work with private entities to facilitate the adoption of a nationwide system for the electronic use and exchange of health information. However, implementation is incomplete; in its most recent (2018) report to Congress, the HHS Office of the National Coordinator for Health Information Technology stated, “[Healthcare] providers often lack access to patient data at the point of care, particularly when multiple [healthcare] providers maintain different pieces of data, own different systems, or use health IT solutions purchased from different developers.”^21^ The shortcomings of the US national health IT infrastructure were also exposed during the COVID-19 pandemic.^22^

Planning, another tool for improving coordination, collaboration, and communication among NDMS partners, was also repeatedly noted as a weakness at the federal and regional levels. The second highest-ranked weakness in the survey was the lack of an overall federal management plan to coordinate the healthcare response across the military, federal, and private sectors. There was an additional opportunity recommended to improve planning at the regional level. These findings are consistent with existing literature on coordinated patient movement and tracking and enhanced collaboration among military, federal, and non-federal civilian healthcare partners.^5^

This survey also identified limited transportation (“patient movement”) and healthcare facility surge capacities as critical weaknesses. Transportation for NDMS patients is the responsibility of the DOD, yet concerns about the available capacities to transport patients nationally between regions and locally within regions during a large-scale event have been raised repeatedly.^5,23-25^

The first year of the COVID-19 pandemic demonstrated the limit of healthcare surge capacity when hospitals across the United States were almost overwhelmed by the unprecedented patient load.^11^ Specific opportunities that survey respondents recommended for increasing hospital surge capacity included: strategic interventions, such as increasing federal funding to hospitals to maintain extra capacity and the inclusion of specialty care sites (eg, mental health, rehabilitation, burn centers) as NDMS patient care partners; and tactical interventions focused on staff, such as increasing the use of telehealth, granting emergency credentials and healthcare license portability, and using Medical Reserve Corps members to provide care.

The issue of NDMS funding has been previously raised^9^ and is invariably tied to many of the opportunities for improvement that were ranked and prioritized in this survey. Two specific high-priority funding opportunities were recommended: to directly fund private sector healthcare providers to maintain additional surge capacity (88%); and to increase the reimbursement for the care of NDMS patients in the event of an activation (90%).

Training, education, and exercises have been repeatedly identified as critical tools to improve healthcare system preparedness,^26,27^ but they continue to be insufficiently addressed.^28^ Survey respondents recommended 7 opportunities for improving training, education, and exercises, although they were generally ranked lower than opportunities related to other themes. The highest priority recommendation in this area (95%) was to improve outreach to private sector healthcare leaders to increase understanding and NDMS participation. The remainder focused on improving federal–private sector exercises for a combat medical surge scenario (92%); just-in-time training for the private sector, emergency medical services, military, and FCC staff; and ongoing civilian training opportunities for military healthcare providers.

The focus of many of the opportunities recommended from the qualitative and quantitative MCNIS research and much of the NDMS literature for almost the last 20 years has been to increase the healthcare coordination capacities at the regional, not federal, level.^5,9,12,13,24,29-33^ ASPR coordinates numerous healthcare preparedness and response programs that focus on regional preparedness and response, including the NDMS,^4^ Hospital Preparedness Program,^34^ Regional Emergency Coordinators,^35^ and Regional Treatment Network for Ebola and Other Special Pathogens.^36^ The Hospital Preparedness Program is the primary source of federal support for healthcare system preparedness and response. It provides funding to state and local health departments for healthcare preparedness resources and to develop healthcare coalitions (HCCs) to coordinate regional preparedness activities. Some researchers have recognized the potential of HCCs to serve as regional healthcare response coordinating bodies,^31,33^ while others have pointed out that most HCCs focus on healthcare system preparedness rather than response.^30^ In 2018, ASPR began directly funding tertiary hospitals to act as central coordinating bodies with the Regional Disaster Health Response System pilot cooperative agreements.^37^ The program aims to strengthen regional healthcare disaster response by improving organization, coordination, and situational awareness; increasing HCC participation; and identifying and developing highly specialized clinical capabilities. There are currently 4 Regional Disaster Health Response System sites: Massachusetts General Hospital (2018), Nebraska Medicine (2018), Colorado/Mountain Plains Regional Disaster Health Response System (2020), and Emory University (2022).^37^

The MCNIS results provide direction for the DOD-based NDMS pilot to supplement ASPR's regional healthcare response efforts. Working in collaboration with interagency partners, the pilot focuses on improving military–federal–civilian coordination at the FCC level in preparation for a large-scale military conflict or catastrophic disaster while facilitating operational improvements to enhance healthcare preparedness and response for any event resulting in mass casualties.

This survey-based study had several limitations. Civilian/private sector stakeholders may have been overrepresented in the survey sample, whereas FCC stakeholders may have been underrepresented, limiting the generalizability of the overall survey results across the NDMS. In addition, group comparisons between respondent types were not possible due to the relatively small sample sizes for each type. Furthermore, this survey was administered during the Delta wave of the COVID-19 pandemic in the United States, which likely impacted the survey response rate.

## Conclusion

The quantitative findings from this survey validated the qualitative results from the earlier MCNIS qualitative discussions. These findings also identified and prioritized specific interventions to improve the capabilities and capacities of the NDMS definitive care component. Primary among these recommended interventions is strengthening regional healthcare response coordination nationwide by increasing NDMS funding and taking other actions to improve IT systems, planning, training, and exercises. These results have informed the development of interventions for improving the NDMS that are to be implemented and evaluated by the pilot at 5 partner sites around the United States over the next few years, with the ultimate goal of saving and improving the quality of life of the military and civilian patients the NDMS serves.
